# Food insecurity and outcomes during COVID-19 pandemic in sub-Saharan Africa (SSA)

**DOI:** 10.1186/s40066-022-00394-1

**Published:** 2022-12-06

**Authors:** Helen Onyeaka, Phemelo Tamasiga, Hugue Nkoutchou, Ashenafi Teshome Guta

**Affiliations:** 1 School of Chemical Engineering, University of Birmingham, Edgbaston, Birmingham, B15 2TT UK; 2 Public Policy in Africa Initiative (PPiAI), Douala, Cameroon

**Keywords:** Food security, COVID-19, Sub-Saharan Africa, Food production, Ordinary least square, Multivariate regression

## Abstract

The outbreak of COVID-19 led to the implementation of lockdowns and social distancing regulations to curb the spread of infections. Consequently, the lockdowns impeded the movement of smallholder farmers, agricultural inputs, and food products thereby disrupting the food supply chains in SSA. Therefore, this paper examines the relationship between food security indicators (accessibility, availability, utilization, stability) and COVID-19. This study uses ordinary least square regression (OLS) models to study the relationship between the food security indicators and COVID-19. The study considers 9 out of 48 sub-Saharan African countries (Benin, Burkina Faso, Cameroon, Chad, Madagascar, Mali, Mauritania, Nigeria, Senegal) due to data availability restrictions. The result of the analysis indicated that a rise in COVID-19 levels negatively impacts all the 4 indicators of food security without exception. This paper underscores the need to consider the disruptions of food security indicators such as diet, nutritional content, access and availability, affordability, and food supply chains. Moreover, the paper discusses mitigating strategies that may alleviate SSA’s food security amidst the COVID-19 pandemic. We recommend that SSA countries invest in quality agricultural and food production infrastructure and supporting industries that contribute directly to the food supply chain, such as agro-processing, fertilizer production and transport. Another important dimension of the COVID-19 and food insecurity syndemic is the income shocks that occurred as a consequence of the COVID-19 outbreak. Like many factories, companies, and service providers closed shop (especially the informal sector), people lost their incomes as a result of loss of employment and, in many instances, no social protection. Therefore, we recommend that SSA governments develop affordable, sustainable, and targeted social protection/insurance systems that extend to the informal sector of the economy.

## Introduction

Food security is one of the pillars of the United Nations 2030 Agenda for Sustainable Development Goals (i.e., SDG #2 Zero Hunger), which serves as a blueprint to achieve a better and more sustainable future for all [16]. The emergence of the novel coronavirus in 2019 (COVID-19) led to the disruption of livelihoods, livestock, agriculture, and supply chains [9, 18]. Ironically, the United Nations, through its specialized agency, the Food and Agriculture Organization (FAO), was presciently preparing for the COVID-19 pandemic when it adopted the theme—safeguarding against economic slowdowns and downturns, emphasizing the importance of protecting nutrition and food security during the crisis in 2019.

Measures (quarantines, lockdowns, restrictions on the movement of goods and people) taken to achieve control of the pandemic have had undesirable effects with significant socio-economic repercussions [6], especially for poor rural farmers in low- and middle-income countries (LMICs) [14]. These socio-economic repercussions of the COVID-19 responsive measures pushed approximately half a billion people into poverty, communities in SSA, North Africa, and the Middle East were hit the hardest [22, 26, 27]. Particularly, sub-Saharan Africa, where half of the countries have poverty rates higher than 35% (if we consider the international poverty line, which is currently $1.90 a day) [20], saw the largest increases in extreme poverty, with an additional 24 million people living below the poverty line due to the pandemic [3].

SSA accounts for about 13% of the global population. Moreover, as suggested above, the number of poor and undernourished people remains high among rural communities [24]. SSA’s vulnerability to the socio-economic impacts of COVID-19 was exacerbated by factors such as poor health facilities, low capacity for testing, timely detection, and response to COVID-19 cases [6, 7]. In addition, the latest figures in Africa show that no less than 50% of the African population is dependent on agriculture as their primary source of livelihood and food [17]. For a continent that relies heavily on rainfed agriculture as the foundation of the economy, the complete and partial lockdown undermined food security and threatened food production as it coincided in many cases with the planting periods [10].

Regarding spending by the agricultural sector, less than 20% of the countries in SSA accomplished their commitments to accelerated agricultural growth and transformation and the Comprehensive Africa Agriculture Development Programme (CAADP) [2]. Expectedly, the COVID-19 pandemic is likely to worsen this situation and the challenges faced by over 1.3 billion Africans [1]. Furthermore, shocking evidence suggested that in less than thirty days into the lockdown, more than half of the households in Malawi, Nigeria, Kenya, and Sierra Leone ran out of food [11, 19]. The closure of schools also amplified the problem as it limited children’s access to school feeding programs across all SSA countries, especially in Nigeria.

The population in Africa is expected to double by 2050, and the demand for food to triple [16]. By discouraging major grain-exporting countries from banning exports, the G20 provided the platform that enabled a smooth flow of grain to the SSA region when India, Russia, Cambodia, and Vietnam lifted the ban on exports [21]. Various SSA countries such as Zimbabwe, Zambia, Rwanda, Tanzania, Kenya, Nigeria, and Malawi ensured food security by increasing grain imports [21, 25]. In addition, these countries also rolled out schemes to support farmer input and assist farmers ahead of the production season, which started in the last quarter of 2020 for most countries [21]. Nonetheless, there have been serious concerns that corruption, poor farmer targeting, and bureaucratic complexities could cripple these farm input subsidies initiatives.

The food security situation in SSA has turned out better than feared, although some risks remain. The higher rainfall during the 2020–2021 summer in southern and eastern regions of SSA is another significant development as it allowed for increased plantings and improved crop production conditions. Interestingly, as estimated by the US Department of Agriculture, there are appreciable prospects of increased maize production in several SSA countries. For example, Zambia, Malawi, Mozambique, Kenya, Tanzania, and Zimbabwe showed prospects of large maize and wheat harvests [25]. These facts suggest an improved harvest for grains and other crops and increased livestock conditions in SSA. On the other hand, the food security situation in SSA could be improved if the African Continental Free Trade Area (AFCFTA) is effectively implemented (and non-tariff barriers reduced) because this will increase competition among farmers and merchants and thus better quality of goods at reduced prices for the consumers [4].

Although SSA has sought solutions to mitigate the devastating impact of the COVID-19 pandemic on food security, this current global stressor has highlighted the need to develop a sustainable intervention to boost food systems in SSA. In this light, this paper seeks to evaluate the implications of COVID-19 on food security in SSA, using observational cases, situational analysis reports, and digital online data sources. We argue that the ongoing debate about the socio-economic impacts of COVID-19 in SSA should focus on, among other things, food security by providing some sustainable recommendations and policy prescriptions to quell food worries in the post-COVID-19 pandemic era.

As suggested above, as of 2020, more than one-third of the population in sub-Saharan was undernourished. In Africa, 282 million people were experiencing hunger, more than double the proportion of any other region in the world. Across East Africa, conditions are deteriorating, with millions of people facing hunger and severe food insecurity. As expected, these levels of food insecurity worsened further in 2021, while a decline in diminished purchasing power, lost livelihoods, income opportunities, and limited access to basic food and services are all continuing into 2022. This situation is exacerbated by the Russia–Ukraine war and its negative effects on food, energy (oil and gas) and fertilizer prices.

The aim of this study is to investigate the relationship between COVID-19 and food security indicators in a sample of sub-Saharan African countries. We restricted the sample countries to 9 sub-Saharan African countries due to the inconsistent data availability of the data points used in the study. Our findings contribute to the growing literature on food security and COVID-19 nexus. Current literature has either used deductive approach to explain how COVID-19 has influenced different food security indicators or provided descriptive statistics on the food security indicators during COVID-19. In addition, some empirical studies have examined the interplay of COVID-19 and the oil and stock market. The gap that our study attempts to fill is to explore how each specific food indicator (accessibility, availability, utilization and stability) relates with COVID-19. The second and most important contribution of this study is that we consider, the relationship between COVID-19 and coronavirus pandemic on developing country subset, whereas many empirical studies have focused on developed countries.

The rest of the paper is structured as follows: theoretical linkages and stylized facts about food security are presented in the next section. Methodology provides the model specification and empirical strategy. Data discusses issues related to data sources and the measurement of variables. The empirical results are presented and discussed in Dependent variables/food security indicators. The last section concludes the paper with the main findings and policy implications.

## Stylized facts on food security and COVID-19

**Fig. 1:**
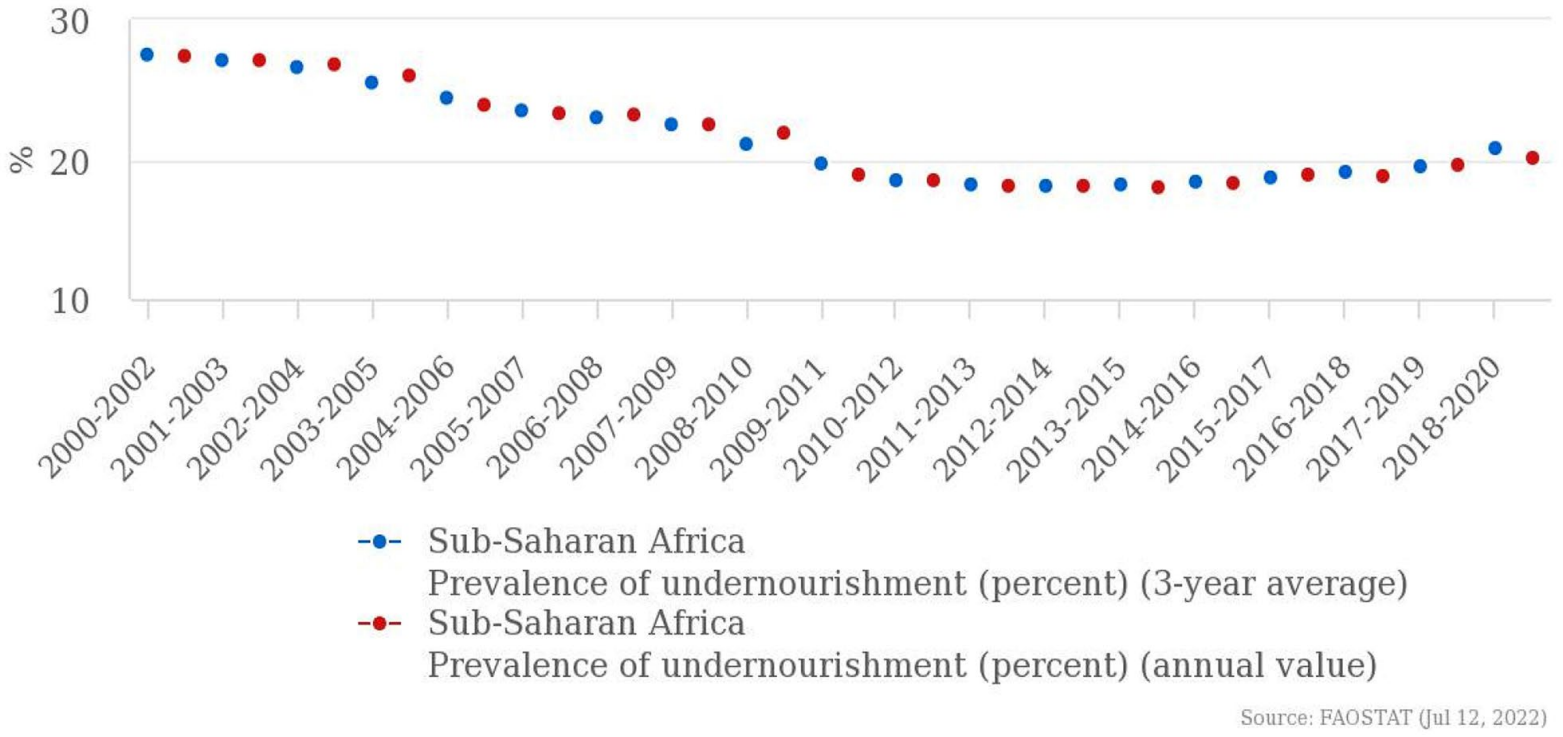
Prevalence of undernourishment (percent) (annual value) Source: FAOSTAT (July 12, 2022)

In disease outbreak events, the severity of the disease also defines the magnitude of the effect on food security. The daily growth rate of confirmed cases of COVID-19 has affected the full front of food security indicators: availability (adequate supply of food), access (the ability of people to get the food they need), and utilization (adequate intake of nutrients) [8]. Access to food has both physical and economic access dimensions. Figure 1 shows that for the sub-Saharan African region there was an increase in the prevalence of undernourishment from 20.2% in 2017–2019 to 21.8% in 2018–2020. This is further illustrated by an increase in severe food insecurity in total population in the region from 55.7% in 2017 to 57.7% in 2019 (see Table 1).

**Table 1:** Prevalence of moderate and severe food insecurity (percent) (annual value) Source: FAOSTAT (July 12, 2022)

Item	Year code	Value (%)
Prevalence of moderate or severe food insecurity in the total population (percent) (annual value)	2014	47.9
Prevalence of moderate or severe food insecurity in the female adult population (percent) (annual value)		54.8
Prevalence of moderate or severe food insecurity in the male adult population (percent) (annual value)		51.2
Prevalence of moderate or severe food insecurity in the total population (percent) (annual value)	2015	49.7
Prevalence of moderate or severe food insecurity in the female adult population (percent) (annual value)		54.5
Prevalence of moderate or severe food insecurity in the male adult population (percent) (annual value)		52.9
Prevalence of moderate or severe food insecurity in the total population (percent) (annual value)	2016	54.2
Prevalence of moderate or severe food insecurity in the female adult population (percent) (annual value)		60.6
Prevalence of moderate or severe food insecurity in the male adult population (percent) (annual value)		59.3
Prevalence of moderate or severe food insecurity in the total population (percent) (annual value)	2017	55.7
Prevalence of moderate or severe food insecurity in the female adult population (percent) (annual value)		64.1
Prevalence of moderate or severe food insecurity in the male adult population (percent) (annual value)		62.7
Prevalence of moderate or severe food insecurity in the total population (percent) (annual value)	2018	55.9
Prevalence of moderate or severe food insecurity in the female adult population (percent) (annual value)		62.3
Prevalence of moderate or severe food insecurity in the male adult population (percent) (annual value)		61.8
Prevalence of moderate or severe food insecurity in the total population (percent) (annual value)	2019	57.7
Prevalence of moderate or severe food insecurity in the female adult population (percent) (annual value)		64.7
Prevalence of moderate or severe food insecurity in the male adult population (percent) (annual value)		62.1

Moreover, the prevalence of moderate or severe food insecurity is further filtered at the level of males and females as shown in Table 1. It can be seen there is high food insecurity among the female population in comparison to the male population from the years 2014–2019. This is an immediate indication that there is a need for gender-differentiated policies that take into consideration the complex link between food security and different demographics of the population.

### Methodology

This section provides an overview of the methodology employed in this study. To quantify the effects of COVID-19 on food security, we model food security Ykt within country k as a function of COVID-19 infections and control variables Xkt. As a point of departure, we specify the econometric model and the dependent and independent variables used:

1 $${\text{Y}}_{{{\text{kt}}}} \, = \,\alpha \, + \,\beta \cdot{\text{COVID}}\_{19}_{{{\text{kt}}}} \, + \,\delta {\text{X}}_{{{\text{kt}}}} \, + \,\mu_{{{\text{kt}}}}$$

where Y_kt_ refers to food security indicators, k refers to a specific sub-Saharan African country and t is the time. COVID-19, refers to the number of COVID-19 infections/cases in a particular country at a particular point in time. The impact of COVID-19 on food security is captured by the parameter β. Control variables are captured by X_kt_. They facilitate the prediction of food security measures. This set includes trade as a percentage of GDP, population, Cereal yield which is a proxy of agricultural productivity which in turn affects food security and GDP per capita.

Equation (1) applied the multivariate Ordinary Least Square (OLS) estimation. We ensured that the requirements of choosing OLS as a suitable estimation method were met. That is; (i) heteroscedasticity test: the standard assumption that the variance of the error term is similar across values of independent variables hence satisfying homoscedasticity. (ii) We checked that there is no persistence of autocorrelation.

### Data

In this section, we provide a summary of the data points used as well as their sources. The pandemic-related data come from Johns Hopkins website. The dataset includes the quantities of COVID-19 infections and covers all sub-Saharan African countries used in this study from 1 January 2020 to 31 December 2021.

The food security data are acquired from Food and Agriculture Organisation (FAO)^1^ The World Development Indicators (WDI) are acquired from the World Bank Website.^2^ A summary of the descriptions and the sources of the data points is provided in Table 2.

**Table 2:** Summary of data points, descriptions and data sources

Variable name	Description	Source
COVID-19 infections	This refers to the COVID-19 confirmed cases at country level	https://raw.githubusercontent.com/Lucas-Czarnecki/COVID-19-CLEANED-JHUCSSE/master/COVID-19_CLEAN/csse_covid_19_time_series_cleaned/time_series_covid19_cases_tidy.csv
Trade as % of GDP	Trade is the sum of exports and imports of goods and services measured as a share of gross domestic product	https://data.worldbank.org/
Population, total	Total population is based on the de facto definition of population, which counts all residents regardless of legal status or citizenship. The values shown are midyear estimate	https://data.worldbank.org/
'GDP per capita (constant 2015 US$)'	GDP per capita is gross domestic product divided by midyear population. GDP is the sum of gross value added by all resident producers in the economy plus any product taxes and minus any subsidies not included in the value of the products	https://data.worldbank.org/
'Cereal yield (kg per hectare)'	Cereal yield, measured as kilograms per hectare of harvested land, includes wheat, rice, maize, barley, oats, rye, millet, sorghum, buckwheat, and mixed grains	https://data.worldbank.org/
Average dietary energy supply adequacy (percent) (3-year average)	The indicator calculates the Dietary Energy Supply (DES) as a percentage of the Average Dietary Energy Requirement (ADER). Each country's or region's average supply of calories for food consumption is normalized by the average dietary energy requirement estimated for its population to provide an index of adequacy of the food supply in terms of calories	FAOSTAT
Prevalence of undernourishment (percent) (3-year average)	The prevalence of undernourishment expresses the probability that a randomly selected individual from the population consumes an amount of calories that is insufficient to cover her/his energy requirement for an active and healthy life	FAOSTAT
Per capita food supply variability (kcal/cap/day)	Per capita food supply variability corresponds to the variability of the "food supply in kcal/caput/day". It compares the variations of the food supply across countries and time	FAOSTAT
Number of children under 5 years affected by wasting (million)	Wasting prevalence is the proportion of children under five whose weight for height is more than two standard deviations below the median for the international reference population ages 0–59	FAOSTAT

### Dependent variables/food security indicators

In order to proxy for food security, it is critical to choose measures that incorporate all the 4 pillars of food security: (1) availability, (2) access (3) stability, and (4) utilization. However, previous empirical literature has mainly studied food availability which they proxied using dietary energy supply [28].

Drawing from the food security indicators from the FAO database, this study followed past studies such as Reddy and Bonuedi and used:

1. average dietary energy supply adequacy as a proxy of food availability,
2. prevalence of undernourishment to proxy accessibility of food,
3. per capita food supply variability to proxy food stability,
4. children affected by wasting to capture food utilization.

This study used these indicators across countries used in this study. In order to reduce the effect of yearly fluctuations in the data, we have taken moving averages of three years (i.e., 2018 to 2020 for the food security indicators). Moreover, we used the moving averages of three years because there were several missing values in the yearly data, a similar approach was employed in the study of Tamasiga et al., [23] who investigated the impact of socio-economic indicators on COVID-19 and Mihoub et al., [12] who applied machine learning techniques in order to make predictions of cases of COVID-19.

### Independent variables

We used the COVID-19 infections as the independent variable. The coefficient of the COVID-19 data point was then used to interpret its interlay with the food security indicators used in this study.

### Control variables

The rationale for the use of the suite of control variables in this study is provided below:

### GDP per capita

It reflects the extent of economic development. Countries with higher GDP per capita have better access to food and better food security for their people.

### Cereal yield

Higher cereal yield reflects the extent of agricultural productivity in a country, hence it can proxy the extent of food security for households.

### Population

Population affects food security through the increase in pressure on the available food resources hence why we considered it part of the control variables. On the other hand, in rural communities, population growth can translate to labor availability for agricultural purposes (food production).

### Trade as a percentage of GDP

According to Dithmer and Abdulai [5], trade has the potential to improve food security status through dietary energy consumption and diversity.

**Table:** 

	Count	Mean	Std	min	25%	50%	75%	max
log trade	9.0	4.07	0.29	3.5	4.1	4.12	4.23	4.41
log population	9.0	16.83	1.0	15.3	16.55	16.76	17.04	19.09
log GDP_pc	9.0	6.96	0.52	6.18	6.58	7.05	7.27	7.83
log cereal_yield	9.0	7.33	0.42	6.79	7.17	7.27	7.41	8.3
log confirmed	9.0	16.23	1.11	14.5	15.49	16.21	16.9	18.14
log prevelance_under_nourishment	9.0	2.41	0.7	1.72	1.97	2.15	2.52	3.74
log average_dietary_energy_supply_adequacy	9.0	4.76	0.13	4.48	4.75	4.82	4.82	4.88
log children_affected_by_wasting	9.0	− 1.28	0.92	-2.3	− 1.61	− 1.2	− 1.2	0.79
log per_capita_food_supply_variability	9.0	3.49	0.46	2.4	3.43	3.5	3.81	3.91

### Descriptive statistics

Table 2 provides a summary of the descriptive statistics of the variables used in the econometric specification.

There are 9 countries considered in this study (Benin, Burkina Faso, Cameroon, Chad, Madagascar, Mali, Mauritania, Nigeria, Senegal). Table 2 reports the mean, max and standard deviations of both the dependent variables and independent variables. We took logarithms of all the data points to standardize it as there were huge variations in the values of the raw/original data points, which might have led to inconsistent or wrong interpretation of the regression results.

### Correlation matrix

**Fig. 2:**
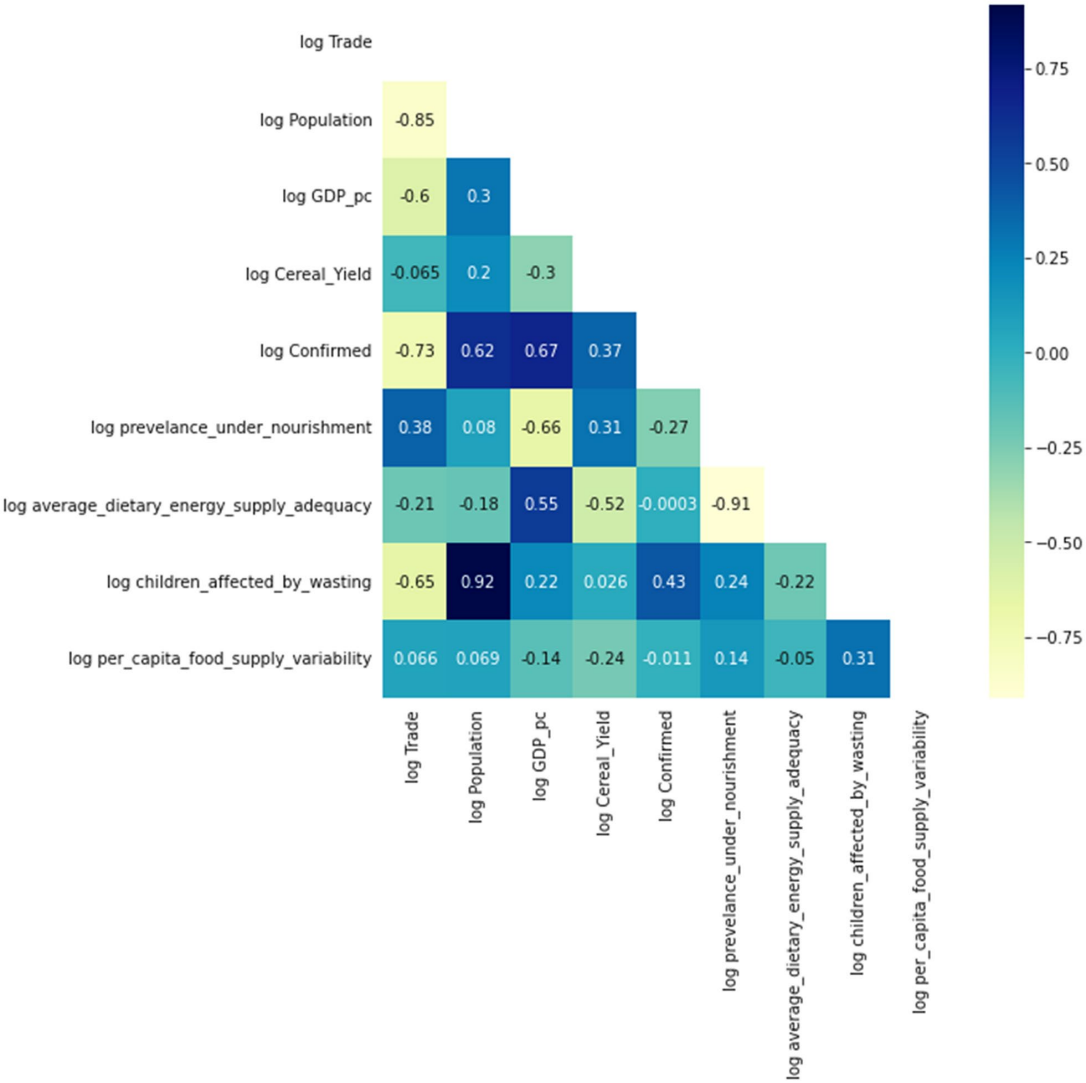
Correlation matrix of variables

Figure 2 displays the heat map, which displays the correlation between the dependent and independent variables. We performed the correlation to check for multicollinearity among the independent variables and with the dependent variable. The rule of thumb for multicollinearity requires that no independent variables should be highly correlated with the dependent variable with a correlation coefficient of − / + 0.80.

**Table 3:** OLS regression results of the prevalence of undernourishment and COVID-19

Dep. variable	log prevalence under-nourishment	R-squared	0.796
Model	OLS	Adj R-squared	0.455
Method	Least squares	F-statistic	2.335
Date	Mon, 11 Jul 2022	Prob (F-statistic)	0.258
Time	22:34:49	Log-likelihood	− 1.8615
No. observations	9	AIC	15.72
Df residuals	3	BIC	16.91
Df model	5		
Covariance type	Nonrobust		

	coef	std err	t	P >|t|	[0.025 0.975]	
const	− 20.5526	16.089	− 1.277	0.291	− 71.755	30.649
log Confirmed	0.2788	0.456	0.611	0.584	− 1.173	1.730
log GDP_pc	− 0.7784	0.868	− 0.896	0.436	− 3.542	1.985
log Trade	3.1529	1.631	1.933	0.149	− 2.037	8.343
log Population	0.7826	0.407	1.922	0.150	− 0.513	2.078
log Cereal_Yield	− 0.2911	0.808	− 0.360	0.743	− 2.864	2.282

Based on Fig. 2, there is no presumed autocorrelation between the food security indicators considered in this study and the level of COVID-19 infections. The correlation coefficient between COVID-19 and the children affected by wasting is 0.43, whereas the prevalence of undernourishment has a correlation of 0.27 with COVID-19. In the next step, we run a regression across all countries to get a clearer picture of the relationship between COVID-19 confirmed cases and the food security indicators.

## Results and analysis

### The relationship between COVID-19 and food accessibility (prevalence of under-nourishment used as a proxy)

Table 3 reports the results obtained when estimating the relationship between food availability (prevalence of undernourishment) and COVID-19. The coefficients estimated indicate a positive relationship between the prevalence of undernourishment and trade. Moreover, the prevalence of undernourishment shows an inverse relationship with cereal yield and GDP per capita, respectively. Regarding the relationship between trade and the prevalence of undernourishment, we see a positive relationship. A plausible explanation is that in sub-Saharan African countries as is the case in most African countries an increase in export production causes a reduction in per capita food production hence an increase in the prevalence of undernourishment. This is in part due to the allocation of more land to the production of the major export crops rather than to food crops as suggested in Sahn, D (1990).

### The relationship between COVID-19 and food stability (per capita food supply variability used as a proxy)

Table 4 shows that an increase in COVID-19 cases is associated with an increase in food supply variability. On the other hand, GDP per capita and the yield of cereals showed a negative relationship with COVID-19. This implies that rising infections are associated with decrease in cereal yields in poor countries. Moreover, both trade and population are associated with a rise in per capita food supply variability.

**Table 4:** OLS regression results of per capita food supply variability and COVID-19

Dep. variable	log per capita food supply variability	R-squared	0.505
Model	OLS	Adj. R-squared	− 0.320
Method	Least squares	F-statistic	0.6118
Date	Mon, 11 Jul 2022	Prob (F-statistic)	0.706
Time	23:09:01	Log-likelihood	− 2.0844
No. observations	9	AIC	16.17
Df residuals	3	BIC	17.35
Df model	5		
Covariance type	Nonrobust		

	coef	std err	t	P >|t|	[0.025 0.975]	
const	6.0605	16.492	0.367	0.738	-46.425	58.546
log Confirmed	0.6623	0.468	1.417	0.252	-0.826	2.150
log GDP_pc	-1.1690	0.890	-1.313	0.281	-4.002	1.664
log Trade	0.8305	1.672	0.497	0.653	-4.490	6.151
log Population	0.0797	0.417	0.191	0.861	-1.248	1.408
log Cereal_Yield	-1.3510	0.829	-1.630	0.202	-3.988	1.286

### The relationship between COVID-19 and food availability (average dietary energy supply adequacy used as a proxy)

Table 5 illustrates that average dietary energy supply adequacy is negatively related with COVID-19. Moreover, rising trade and rising population are negatively associated with the average dietary energy supply. This is suggesting that export crops are giving preference over food crops as it is the case in some countries in sub-Saharan Africa (cocoa, coffee, etc.). On the other hand, cereal yield and GDP per capita are positively related with the average energy supply.

**Table 5:** OLS regression results of average dietary supply adequacy and COVID-19

Dep. variable	log average dietary energy supply adequacy	R-squared	0.718
Model	OLS	Adj. R-squared	0.249
Method	Least squares	F-statistic	1.530
Date	Mon, 11 Jul 2022	Prob (F-statistic)	0.386
Time	23:12:48	Log-likelihood	12.074
No. observations	9	AIC	− 12.15
Df residuals	3	BIC	− 10.96
Df model	5		
Covariance type	Nonrobust		

	coef	std err	t	P >|t|	[0.025 0.975]	
const	8.4801	3.420	2.479	0.089	− 2.405	19.365
log confirmed	− 0.0794	0.097	− 0.819	0.473	− 0.388	0.229
log GDP_pc	0.1625	0.185	0.880	0.444	− 0.425	0.750
log trade	− 0.4556	0.347	− 1.314	0.280	− 1.559	0.648
log population	− 0.1068	0.087	− 1.234	0.305	− 0.382	0.169
log cereal_yield	0.0119	0.172	0.070	0.949	− 0.535	0.559

### The relationship between COVID-19 and food utilization (children affected by wasting used as a proxy)

In Table 6, it can be seen that a rising number of COVID-19 cases is associated to a rise in the proportion of children affected by wasting. On the other hand, a rise in cereal yield is associated with a decline in the proportion of children affected by wastage. This empirical result underlines the importance of cereals yield for food utilization since the daily nutritional requirements are mainly extracted from cereal consumption. A rise in trade and population is associated with a rise in children affected by wastage. The latter is in line with our previous comment on export crops being given priority over food crops in some countries in Africa.

**Table 6:** OLS regression results of children affected by wasting and COVID-19

Dep. variable	log children_affected_by_wasting	R-squared	0.951
Model	OLS	Adj. R-squared	0.869
Method	Least squares	F-statistic	11.63
Date	Mon, 11 Jul 2022	Prob (F-statistic)	0.0353
Time	23:15:48	Log-likelihood	2.0996
No. observations	9	AIC	7.801
Df residuals	3	BIC	8.984
Df model	5		
Covariance type	Nonrobust		

	coef	std err	t	P >|t|	[0.025 0.975]	
const	− 30.0464	10.361	− 2.900	0.062	− 63.018	2.926
log Confirmed	0.0426	0.294	0.145	0.894	− 0.892	0.977
log GDP_pc	0.1025	0.559	0.183	0.866	− 1.677	1.882
log Trade	2.0731	1.050	1.974	0.143	− 1.269	5.415
log Population	1.3432	0.262	5.123	0.014	0.509	2.178
log Cereal_Yield	− 0.5020	0.521	− 0.964	0.406	− 2.159	1.155

## Discussion

To gain valuable insights into the relationship between COVID-19 and food security in SSA, this study, employed proxies of food security indicators and performed a multivariate regression. The R^2^ value of food availability (0.718), accessibility (0.796), stability (0.505), and utilization (0.951), means that the variation in the values of independent variables can be explained by 71.8% (for the food availability equation), 79.6% (for the food accessibility equation), 50.5% (for the food stability equation) and 95.1% (for the food utilization equation) by the variation of the dependent variable.

### Food accessibility

The most vulnerable members of society, such as those with low economic status especially in rural settlements, the disabled, and the elderly, are most likely to suffer from the inaccessibility of food with standard daily nutritional requirements amid the COVID-19 pandemic. As shown Table 3, COVID-19 is associated with a rise in the prevalence of undernourishment. Since we used undernourishment as a proxy of food Accessibility, this means that COVID-19 has a worsening effect on food accessibility. Food accessibility has been hampered by border restrictions, lockdowns, and job losses. Due to extended transportation periods, food and livestock sellers have seen an increase in damaged commodities. In East Africa, truckers have often queued for miles and endured lengthy delays delivering goods to market. Many people have been unable to flee across the border into neighboring countries in eastern DRC, the region with the continent's biggest forcibly displaced population. Cities in Africa have always been excluded from food security assessments since they have historically been better off than rural areas. As a result of income-depriving restrictions imposed in the aftermath of COVID-19, several cities' disadvantaged populations were subjected to severe food insecurity.

### Food availability

The OLS results displayed in Table 5 showed that the average dietary energy supply adequacy was negatively associated with COVID-19 cases. This means that as COVID-19 cases rise, the energy supply adequacy declines. Hence, we can interpret from this proxy, that COVID-19 is associated with a decline in food availability. Supply chain disruptions impact food items' prices due to the supply and demand shocks emanating from closed borders and restricted movement, which impact the transportation of food items within countries and across borders. Price increases in food items consumed by households led to a decrease in purchasing power. To get a vivid picture of the impact of COVID-19 on food availability, it is also crucial to understand the dynamics of the baseline income and food consumption trends in sub-Saharan Africa. The intervention of the government to provide food hampers is low in many of the sub-Saharan countries. The lack of governmental intervention in poor sub-Saharan African countries further exacerbated the food insecurity challenge during COVID-19. Acute food emergencies in Africa are still mostly caused by conflict. Famine is now affecting parts of South Sudan (Northern Bahr El Ghazal, Jonglei, and Warrap States) and Burkina Faso (Soum and Oudalan Provinces) (Phase 5). Conflict-affected communities can also be found in the Democratic Republic of the Congo (DRC), Ethiopia, Mali, Niger, Nigeria, and Cameroon. These nations are experiencing active conflicts as a result of militant Islamist group strikes in the Sahel and ongoing terrorist attacks on roads, particularly in northeast Nigeria, which are restricting food and humanitarian access. As a result of the ongoing violence in Ethiopia, food insecurity is particularly high in Tigray, while most of the territory remains inaccessible to UN and relief agencies. Due to rising Boko Haram assaults in Cameroon's Far North Region and separatists and military conflicts in the country's Anglophone Northwest and Southwest Regions, Cameroon has a significant food crisis. In the Central African Republic, renewed fighting has resulted in a deliberate rebel blockage of food and humanitarian aid from reaching Bangui.

### Food utilization

According to Table 6, an increase in COVID-19 cases is associated with a rise in the proportion of children affected by wasting. This in turn implies that COVID-19 is associated with worsening food utilization. The possible consequence of COVID-19 and food insecurity is that children who are still growing may suffer from growth and developmental impairment as a result of nutritional inadequacies. In many SSA schools, pupils are provided with a basic meal, school closures may lead to nutritional inadequacies as many of the children come from poor economic backgrounds, and this dietary inadequacy may lead to low educational achievement, cognitive deficits, chronic physical and mental health problems, and starvation [15]. Moreover, skipping meals due to financial constraints, hoarding food, and pressuring can negatively affect child wellbeing [15].

### Food stability

In Table 4 we used the per capita food supply variability as a proxy for food stability. The table shows that when COVID-19 rises, the per capita food supply variability also rises. The interpretation of this result is that COVID-19 results in food supply variability which is a consequence of changes in production, trade, government policies and distribution. Furthermore, food supply variability, as reflected in food price volatilities makes it difficult for households to budget for food within the confines of their financial resources. Therefore, we can deduce that COVID-19 negatively impacts food stability.

In principle, COVID-19 affects all 5 phases of the food supply chain: agricultural production, postharvest handling, processing, distribution/retail/service, and consumption [13]. In SSA countries, a large percentage of farm produce was not able to reach markets due to movement restrictions and border closures, leading to large losses of agricultural producers. The movement restrictions during the COVID-19 coincided with planting periods for most of the staple crops and reduced the quantities produced by farmers [2]. As discussed above, the food supply chain shock caused by a closure of borders led to a reduction in import quantities, thereby a rise in the prices of imports, especially perishables. Retail stores (grocery supermarkets) were often vacant, which led to a lack of essential food items. Another element ignored by most literature is that movement restriction stopped farmers in developing countries from having access to agricultural advice services and training from government agricultural units and departments. Over and above the supply and consumption dimensions of the supply chain, movement restrictions and social distancing protocols limited labor availability which is an important input into the agricultural and food processing industry. In many sub-Saharan African countries, a large proportion of the population works in the informal sector, and closure of business meant that informal markets for small farm holders were shut down and this is a direct impact on their income levels and on the physical access to food by the communities in which they operate.

## Conclusions and recommendations

This study aimed to investigate the linkage between food security indicators and COVID-19 in a subset of sub-Saharan African countries. Studying and understanding the impacts of COVID-19 on food security is critical to achieving the sustainable development goal of ending hunger and improving and promoting agricultural sustainability and nutrition. Therefore, we employed a multivariate OLS regression model. In General, we found out that COVID-19 is negatively associated with all the food security indicators considered in this study. We, therefore, draw the following recommendations for SSA countries:

- Deliberate attempts need to be made to establish well-funded food and agricultural data collection and analysis hubs that will inform policies geared towards ameliorating COVID-19 impacts on food availability and gaining insights into inter-connections between SSA regions and across agricultural produce.
- SSA governments need to establish targeted food insecurity insurance packages at affordable rates, especially for the most vulnerable members of the community. Also, deliberate strategies should be put in place to include the underprivileged older adults and those living below the poverty line living mostly in the rural areas.
- A large proportion of the labor force in SSA countries is informal sector workers. Governments should improve their social security packages to be extended to the informal sector while maintaining sustainability of public finance. As discussed above, social distancing and movement restrictions led to massive employment losses in the informal sector, which stemmed from business closures. The income shock was exacerbated in part by the lack of social security for this sector.
- COVID-19 has exposed the inherent fragilities of SSA agricultural and food supply chains. Therefore, there is a need to increase the resilience of SSA supply chains to future shocks emanating from pandemics or natural disasters. SSA governments and the private sector should compete fairly in transforming the agriculture sector and endeavor to develop quality infrastructure, long-term storage facilities for fresh produce, and promote innovation of the agricultural industry. Strong contract enforcement measures will help unleash the potential of the private sector. In other words, over and above the government incentives to stimulate agricultural production, we recommend private sector participation and investment in agricultural technology to cushion food prices during pandemics.
- The food production industry is intertwined with suppliers in industries that support the food supply chain. In a bid to improve the food security situation, SSA countries should deliberately create an enabling environment that supports these industries and develop non-agriculture infrastructure that supports the food supply chain such as market access, transport (road and rail), logistics infrastructure, electricity, and e-commerce solutions for payments to name a few.
- Land devoted to food crops should not be used for export crops, as this can have a negative effect on local food availability.

## Data Availability

Not applicable.

## Footnotes

1 FAOSTAT

2 WDI—Home (worldbank.org).
